# Transcriptome analysis of brassinolide under low temperature stress in winter wheat

**DOI:** 10.1093/aobpla/plad005

**Published:** 2023-02-02

**Authors:** Meiyun Ding, Luyao Wang, Yuting Sun, Junbao Zhang, Yushu Chen, Xuesong Wang, Lijie Liu

**Affiliations:** College of Life Science and Agriculture Forestry, Qiqihar University, 42 Wenhua street, Qiqihar 161006, Heilongjiang, China; College of Life Science and Agriculture Forestry, Qiqihar University, 42 Wenhua street, Qiqihar 161006, Heilongjiang, China; College of Life Science and Agriculture Forestry, Qiqihar University, 42 Wenhua street, Qiqihar 161006, Heilongjiang, China; College of Life Science and Agriculture Forestry, Qiqihar University, 42 Wenhua street, Qiqihar 161006, Heilongjiang, China; College of Life Science and Agriculture Forestry, Qiqihar University, 42 Wenhua street, Qiqihar 161006, Heilongjiang, China; College of Life Science and Agriculture Forestry, Qiqihar University, 42 Wenhua street, Qiqihar 161006, Heilongjiang, China; College of Life Science and Agriculture Forestry, Qiqihar University, 42 Wenhua street, Qiqihar 161006, Heilongjiang, China

**Keywords:** Brassinolide, cold resistance, transcriptome, *Triticum aestivum*

## Abstract

Low temperatures are the main abiotic factor affecting wheat growth. Brassinolide is a novel plant hormone that can improve the cold resistance of plants; however, the molecular mechanism of brassinolide in winter wheat at low temperatures remains unclear. In this study, winter wheat Dongnong dongmai 1 was sprayed with 0.01, 0.1, or 1.0 mg·L^–1^ brassinolide (BR) at the three-leaf stage, and tillering nodes were sampled at different temperatures (5, –10 and –25 °C), and then physiological indexes were determined and the transcriptome was sequenced. The results showed that the optimum concentration of brassinolide for cold resistance is 0.1 mg·L^–1^. A total of 15 302 (8198 upregulated and 7104 downregulated) differentially expressed genes (DEGs) were identified in the B1 vs D1 comparison (B1 represents 5 °C 0.1 mg·L^–1^ BR treatment, D1 represents 5 °C control); 3386 (1930 upregulated and 1456 downregulated) differentially expressed genes (DEGs) were identified in the B2 vs D2 comparison (B2 represents –10 °C 0.1 mg·L^–1^ BR treatment, D2 represents –10 °C control); and 2684 (2102 upregulated and 582 downregulated) differentially expressed genes (DEGs) were identified in the B3 vs D3 comparison (B3 represents –25 °C 0.1 mg·L^–1^ BR treatment, D3 represents –25 °C control). Further studies showed that these DEGs were mainly involved in carbon fixation in photosynthetic organs, photosynthesis and plant–pathogen interactions, all of which were related to stress and energy metabolism. This indicates that brassinolide can produce substances that improve cold resistance in wheat seedlings. This study provides a theoretical basis for further research on the improvement of cold resistance in winter wheat by brassinolide.

## Teaser

In this paper, the molecular mechanism of the effect of brassinolide on the cold resistance of winter wheat under different low temperatures was explored. Transcriptomics were used to analyse the effect of brassinolide on the expression of genes in the tillering nodes of winter wheat Dongnong dongmai 1. The analysis concluded that brassinolactone can enhance the metabolism of winter wheat, and produce more energy and material resistance to or adapt to the adverse environment under different cold temperatures. These results expand our understanding of the molecular mechanisms by which brassinolide affects cold resistance in winter wheat, and this provides a valuable resource for further studies of the effects of brassinolide on Dongnong dongmai 1 under cold stress.

## Introduction

Low temperatures are one of the main factors affecting the growth and development of plants, destroying plant growth, and even causing the death of plants (Levitt 1980). Plant hormones can induce stress tolerance in various plants. Brassinosteroids are a class of plant hormones that regulate a wide range of biological processes, thereby improving plant tolerance to various stressors (Nawaz *et al.* 2017).

Wheat is the main food crop in China and one of the main food crops grown worldwide. Wheat is characterized by a high-yield potential and multidirectional use of grains. In the alpine regions of China, the climate is severe in winter, with the lowest temperature reaching approximately –30 °C, which limits the planting of crops. Dongnong dongmai 1 is the first winter wheat variety that can survive the winter in the alpine region of Heilongjiang Province, China, with a greening rate as high as 85 %. However, if it is difficult to store snow in the field or the amount of snow is low, some wheat seedlings will emerge from the snow, and the rejuvenation rate will decrease in the following year. Understanding the molecular mechanisms of crop responses to low-temperature stress is an important means to solve these problems.

Transcriptome analysis using next-generation sequencing technology can detect the molecular mechanisms underlying plant responses to abiotic stress (Wang *et al.* 2009). Some studies have already been carried out on the response of brassinolide (BR) to low-temperature stress in plants (Yang *et al.* 2019). Brassinosteroids are steroid hormones that are synthesized from the bulk sterol campesterol through multiple hydroxylation and oxidation events, which are catalyzed by different cytochrome P450 enzymes, including DWARF4 (DWF4), CONSTITUTIVE PHOTOMORPHOGENESIS AND DWARFISM (CPD), ROTUNDIFOLIA 3 (ROT3), and the CYP85A2 BR6ox2 (Clouse 2011). For studies in the model plant *Arabidopsis thaliana*, it was demonstrated that plants of the BR-supermarker line exhibited higher freezing tolerance at –10 °C compared with the wild type, with disrupting mutations in BR signalling resulting in significantly reduced frost tolerance (Eremina *et al.* 2016). Treatment of pepper seedlings with BR increases tolerance to cold stress, and transcriptome analysis has shown that BR upregulates the expression of thousands of genes in pepper under cold stress (Li *et al.* 2016). BR activates C repeat binding factor (CBF), a key transcription factor responsible for the regulation of genes that respond to low-temperature stress (Anwar *et al.* 2018).

However, there are few studies on the response of brassinolide to different low-temperature stresses in winter wheat. Under freezing stress, external application of epibrassinolide (EBR) can improve the activity of antioxidant enzymes in wheat plants, increase their ability to scavenge reactive oxygen species and reduce the degree of membrane lipid peroxidation (Zhang *et al.* 2022). Treating wheat seedlings with 0.1 mg/L EBR can increase the activities of superoxide dismutase (SOD), peroxidase (POD) and catalase (CAT) in wheat, significantly increase the soluble sugar and Pro content, and decrease the MDA content, thereby improving the frost resistance of wheat (Liu *et al.* 2020).

Here, we first confirm that BR can improve the cold resistance of winter wheat in the seedling and overwintering stages, but the specific molecular mechanism for this requires further study. Therefore, in this study, the transcriptome was used to analyse the response of brassinolide to low-temperature stress in wheat. Transcriptome mapping provided more information about BR-related gene sequences in wheat under different temperatures and the identification of DEGs under low-temperature stress. These results reveal the molecular mechanism by which brassinolide regulates cold resistance in winter wheat.

## Materials and Methods

### Material

Dongnong dongmai 1, a strong cold-resistant wheat variety, was selected as the experimental material. Seeds of Dongnong dongmai 1 with large and full grains were selected and sown in the experimental field. At the three-leaf stage of winter wheat, 0.01, 0.1 or 1.0 mg·L^–1^ brassinolide was sprayed on the leaves and the control group was sprayed with distilled water. Tillering nodes were collected at different temperatures (average minimum temperature for ten consecutive days) (5, –10 and –25 °C) during the overwintering period. The sowing date was September 8, 2021, with artificial planting in a complete block design, with three repetitions, row length 2.0 m, row spacing 0.5 m, 200 seeds per row, sowing depth 5 cm, combined application of nitrogen, phosphorus and potassium fertilizers, diammonium 23 g·m^–2^ and potassium sulfate 7.5g·m^–2^, and conventional field management. Three biological replicates were set for each group. The first sampling was carried out on October 15, 2021, and the average minimum temperature for 10 consecutive days was 5 °C. The second sampling was carried out on December 4, 2021, and the average minimum temperature for 10 consecutive days was –10 °C. The third sampling was carried out on December 31, 2021, and the average minimum temperature was –25 °C for 10 consecutive days. Fifteen tillering nodes were collected from the control group and the treatment group, respectively, and quick-frozen in liquid nitrogen, marked clearly and stored at –80 °C.

### Temperature change during winter

We monitored the outdoor temperature over the winter. The overall average minimum temperature in October 2021 was 2 °C, and the average maximum temperature was 12 °C. The first sampling date was October 15, 2021, and the average minimum temperature for 10 consecutive days was 5.7 °C. In November 2021, the overall average minimum temperature was –5 °C, and the average maximum temperature was 3 °C. In December 2021, the overall average minimum temperature was –15 °C, and the average maximum temperature was –6 °C. The second sampling date was December 4, 2021, and the average minimum temperature for 10 consecutive days was –10.2 °C. The third sampling date was December 31, 2021, and the average minimum temperature for 10 consecutive days was –25.2 °C. In January 2022, the overall average minimum temperature was –17 °C, and the average maximum temperature was –8 °C. In February 2022, the overall average minimum temperature was –18 °C, and the average maximum temperature was –7 °C (Table 1).

**Table 1.** Sequencing data quality statistics. Note: B1: 5 °C treatment group; B2: –10 °C treatment group; B3: –25 °C treatment group; D1: 5 °C control group; D2: –10 °C control group; D3: –25 °C control group.

Sample	raw_reads	clean_reads	clean_bases	error_rate	Q20	Q30	GC_pct
D1	79342477	78776032	11.82G	0.04	97.22	92.02	53.01
D2	90619005	90032505	13.50G	0.03	97.84	93.21	53.46
D3	75639447	74915467	11.24G	0.03	97.65	92.93	52.09
B1	83991477	83592932	12.54G	0.03	98.07	93.79	51.83
B2	79028477	78553496	11.78G	0.03	97.91	93.39	52.36
B3	87284296	86599589	12.99G	0.03	97.93	93.40	52.59

### Measurement of physiological indexes of winter wheat

Malondialdehyde (MDA) content was measured using the TBA colorimetric method (Cang *et al.* 2013), peroxidase activity (POD) using the colorimetric method (Li *et al.* 2009), superoxide dismutase activity (SOD) using the NBT chromogenic method and catalase activity (CAT) was titrated using potassium permanganate (Wang and Huang 2006).

### RNA extraction and cDNA library construction

RNA was extracted using a Tiangen Polysaccharide Polyphenol Kit (Qiagen, Germany) following the manufacturer’s instructions. Accurate detection of RNA integrity and total amount was achieved using an Agilent 2100 bioanalyser. The starting RNA for library construction was total RNA, and the total amount was ≥1 µg. PolyA-tailed mRNAs were enriched using oligo (dT) magnetic beads, and the resulting mRNAs were then randomly disrupted with divalent cations in fragmentation buffer. Using fragmented mRNA as a template and random oligonucleotides as primers, the first strand of cDNA was synthesized in the M-MuLV reverse transcriptase system, the RNA strand was degraded by RNase H, dNTPs were used as the DNA polymerase I system, and the second strand of cDNA was synthesized from dNTPs. The purified double-stranded cDNA was end-repaired, A-tailed, and ligated to sequencing adapters. The cDNA of approximately 370–420 bp was screened with AMPure XP beads, amplified by PCR and the PCR product was purified again with AMPure XP beads to finally obtain the library. After the library had been prepared using a Qubit2.0 Fluorometer for preliminary quantification, the library was diluted to 1.5 ng/µL, and the insert size of the library was determined using an Agilent 2100 bioanalyser by qRT-PCR (higher than 2 nM) to ensure library quality.

### Transcriptome sequencing and assembly

After checking the quality of the libraries, the different libraries were pooled according to the requirements of effective concentration and target data volume. Illumina NovaSeq 6000 sequencing was performed, and 150 bp paired-end reads were generated. The basic principle of sequencing is sequencing during synthesis. Four fluorescently labelled dNTPs, DNA polymerase, and adapter primers were added to the sequencing flow cell for amplification. When the complementary chain was extended by each sequencing cluster, the corresponding fluorescence was released by each fluorescently labelled dNTP, and the fluorescent signal was captured by the sequencer. The light signal was then converted into a sequencing peak using computer software to obtain the sequence information of the fragment to be detected.

### Annotation and functional enrichment analysis of differentially expressed genes

Gene annotation and functional enrichment analyses, including Gene Ontology (GO) and Kyoto Encyclopedia of Genes and Genomics (KEGG) biological pathways, were used to identify which DEGs of wheat tillering nodes were significantly enriched in GO terms or biological pathways after low-temperature and brassinolide treatment. GO enrichment analysis of DEGs was performed using clusterProfiler R software, and GO terms with a corrected *P*-value of <0.05 were regarded as significantly enriched. The statistical enrichment of differentially expressed genes in the KEGG pathway was analysed using clusterProfiler R software. Blast (version: v2.2.23) software was used to compare the measured UniGene sequence with NR (non-redundant protein sequence database, ftp://ftp.ncbi.nih.gov/blast/db), Swiss-prot (http://web.expasy.org/docs/swiss-prot_guideline.html),Pfam(http://pfam.xfam.org/), COG (Clusters of Orthologous Groups of proteins, http://www.ncbi.nlm.nih.gov/COG/), GO (Gene Ontology, http://geneontology.org/) and KEGG (Kyoto Encyclopedia of Genes and Genomes, http://www.genome.jp/kegg/). Six databases were compared, KEGG orthography was analysed by kobas 2.0 software, and the Hmmer (E-va 1e-10) software was then compared with the Pfam database to obtain the UniGene annotation information.

### Screening and correlation analysis of differentially expressed genes

For samples with biological replicates, differential expression analysis between the treatment and control groups was performed using DESeq2 software (1.20.0). The hormone-treated groups B1, B2 and B3 were compared with their respective control groups D1, D2 and D3, and the raw data obtained by sequencing contained a small number of reads with sequencing adapters or low sequencing quality. To ensure the quality and reliability of data analysis, it was necessary to filter the original data. This mainly entailed removing reads with adapters, removing reads containing N (N means that the base information cannot be determined), and removing low-quality reads (the number of bases with Qphred ≤ 20 accounts for more than 50 % of the entire read length). To determine whether the difference in expression levels between the two samples was due to various errors or an essential difference, hypothesis testing on the expression data of all genes in the two samples was needed. Transcriptome analysis was performed on thousands of genes, leading to the accumulation of false positives. The higher the number of genes, the higher the accumulation of false positives in hypothesis testing. The FC (fold change) algorithm was used to identify differences in gene expression levels under two different experimental conditions. The principle of the algorithm is to calculate the fold value of the average expression level of the gene in the two types of samples, and then determine the gene as a differential expression gene. Therefore, padj was introduced to analyse the *P*-value of hypothesis testing, thus controlling the proportion of false positives with |log2(FoldChange)| ≥ 1 & padj ≤ 0.05.

### qRT-PCR validation of gene expression

**Figure 1.**
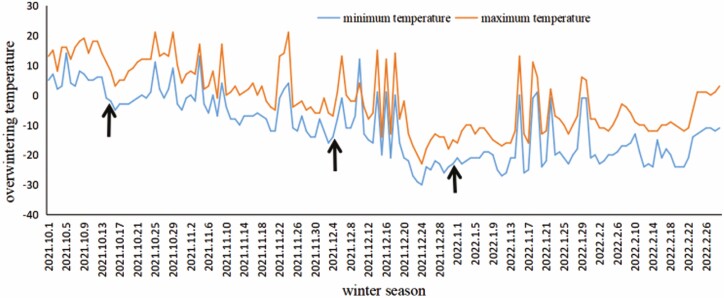
Overall temperature variation trend during wintering. Note: The arrows in the figure represent the sampling dates.

For the wheat transcriptome data, six genes were selected from the differential genes for qRT-PCR verification. The internal reference gene β-actin was used as a control for data normalization to correct for quantitative differences in cDNA used as the template. Fluorescent quantitative PCR primers were designed, total leaf RNA was extracted, and an M-MuLV first-strand cDNA synthesis kit was used for first-strand cDNA synthesis after detection. qRT-PCR was performed using a fluorescence PCR quantifier. Relative gene expression analysis was performed using the 2–∆∆CT method (Fig. 1).

## Results

### Effects of brassinolide on membrane damage in winter wheat tillering nodes at low temperature

**Figure 2.**
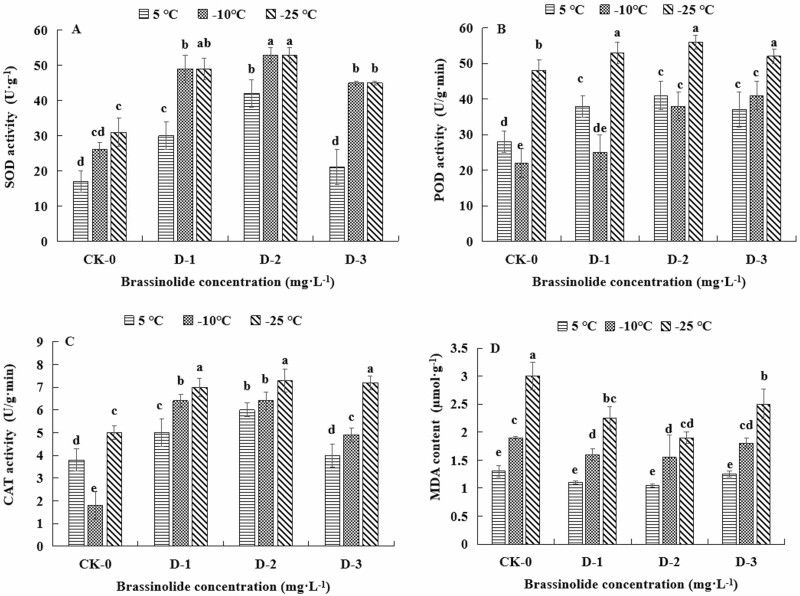
Effects of brassinolide on membrane quality and antioxidant enzyme activities in winter wheat tillering nodes. Note: The different letters indicate significant differences (*s* < 0.05). CK:Dongnong dongmai 1; 1–3:BR concentration (0.01, 0.1 and 1 mg/L).

In both the control groups and the BR treatment groups, the MDA content in the tillering nodes of Dongnong dongmai No. 1 gradually increased as temperature decreased. As the BR concentration increased, the MDA content showed a trend of first decreasing and then increasing and was always lower in the treatment group than in the control group. The degree of membrane damage in Dongnong dongmai 1 was lowest at the BR concentration of 0.1 mg·L^–1^. Compared with the control, the difference was significant (*P* < 0.05) (Fig. 2D).

#### Effects of brassinolide on antioxidant enzyme activities in winter wheat tillering nodes at low temperature.

 In the control groups and BR treatment groups, the SOD activity of Dongnong dongmai 1 showed an upward trend as temperature decreased, and the CAT activity showed a decreasing and then increasing trend. As the BR concentration increased, the overall SOD activity first decreased and then increased. The overall CAT activity first increased and then decreased, and was always higher in the treatment group than in the control group. The highest SOD content and CAT activity in Dongnong dongmai 1 was recorded at the BR concentration of 0.1 mg·L^–1^ (Fig. 2A,C).

As temperature decreased, the POD activity of Dongnong dongmai 1 first decreased and then increased. As the BR concentration increased, POD activity first increased and then decreased, and was always higher in the treatment group than in the control group. POD activity was highest at the BR concentration was 0.1 mg·L^–1^ (Fig. 2B).

### Data evaluation of the transcriptome of Dongnong dongmai 1

**Figure 3.**
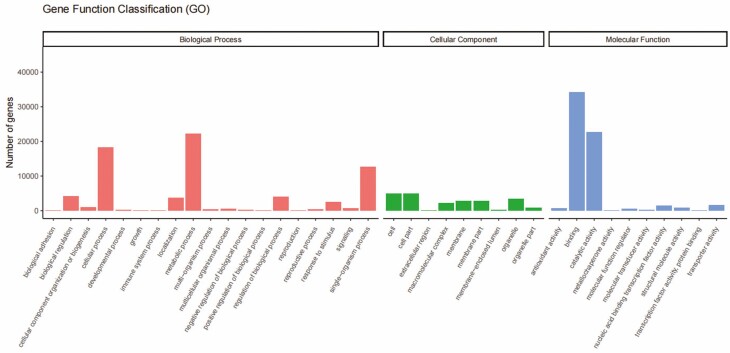
The GO analysis of the differentially expressed genes. Note: GO taxonomy of assembled single genes in wheat tillering nodes.

**Figure 4.**
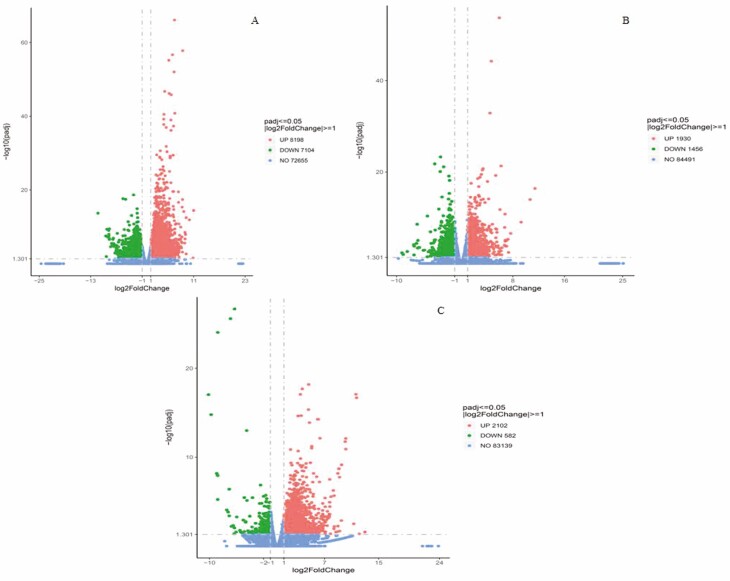
Volcano plots of combined differentially expressed genes. Note: A, B1 vs D1; B, B2 vs D2; C, B3 vs D3.Volcano plots of DEGs between control groups (D1, D2, D3) and treatment groups (B1, B2, B3). The abscissa represents the fold change (log2FoldChange) of gene expression in the treatment and control groups, and the ordinate represents the significance level (–log10padj or –log10pvalue) of the difference in gene expression between the treatment and control groups. The upregulated genes are indicated by red dots, the downregulated genes are indicated by green dots. Genes that were not significantly up- or downregulated are indicated by blue dots.

**Figure 5.**
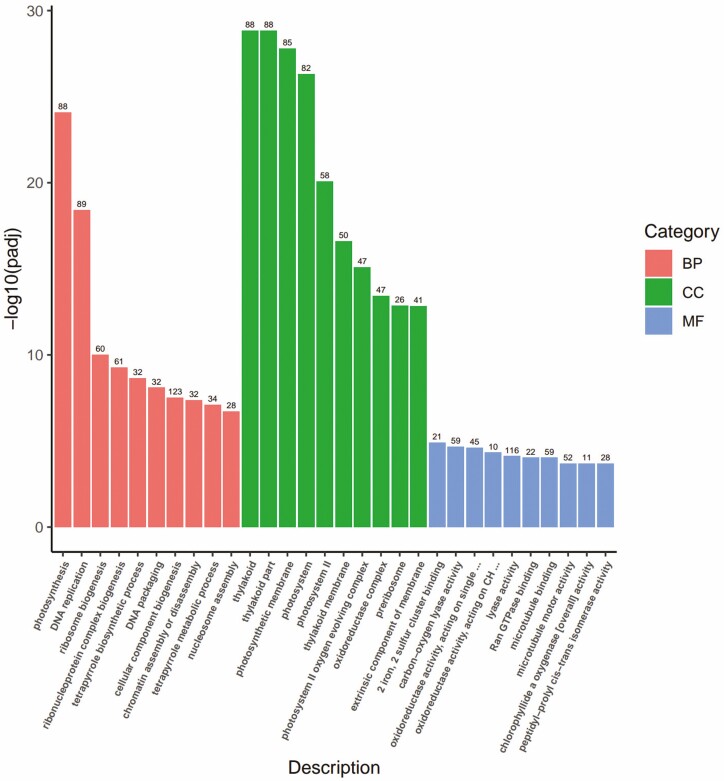
B1 vs D1 Gene Ontology (GO) enrichment analysis of differentially expressed genes (DEGs).

**Figure 6.**
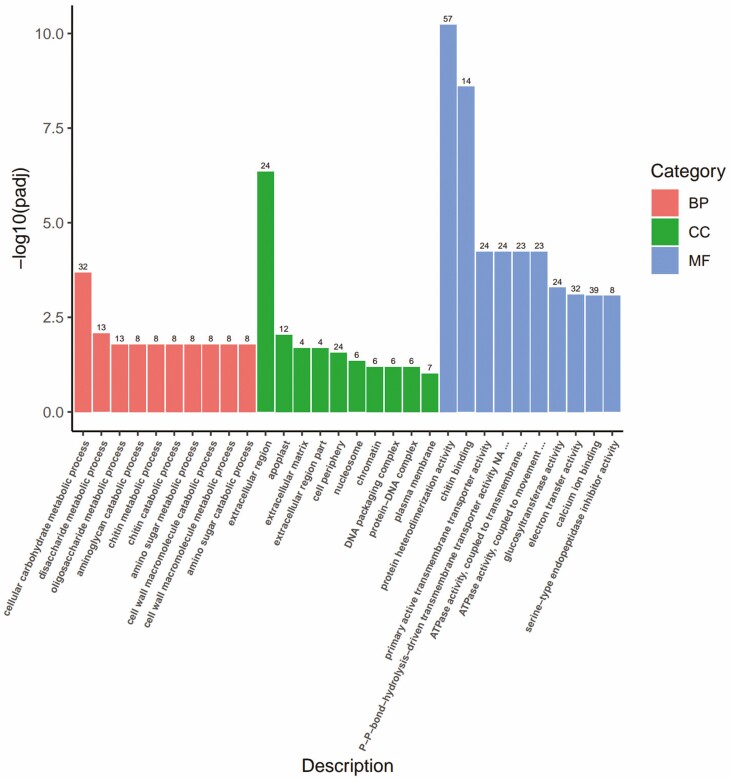
B2 vs D2 Gene Ontology (GO) enrichment analysis of differentially expressed genes (DEGs).

**Figure 7.**
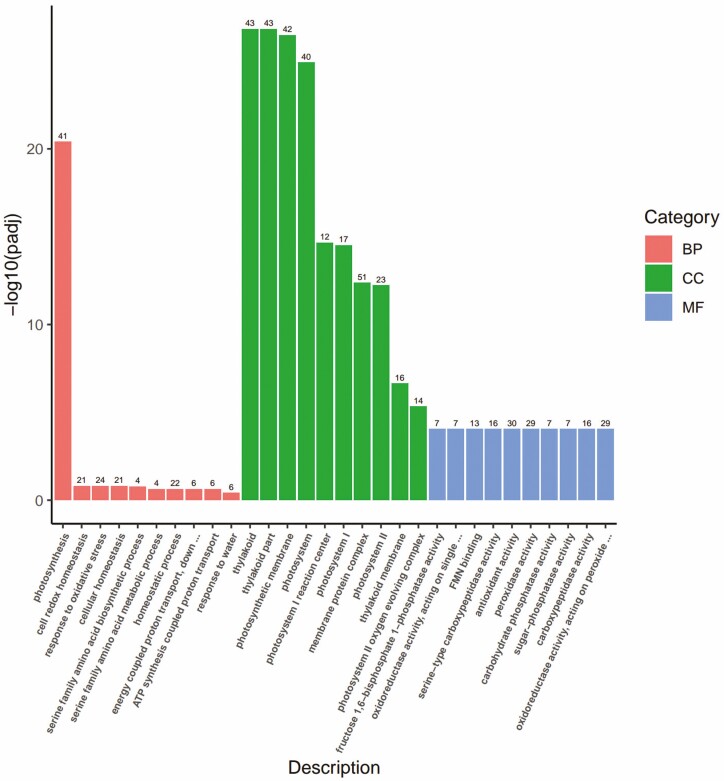
B3 vs D3 Gene Ontology (GO) enrichment analysis of differentially expressed genes (DEGs).

A cDNA library of BR treatment groups (B1, B2 and B3) and their respective control groups (D1, D2 and D3) of Dongnong dongmai 1 at the different temperatures (5, –10 and –25  C, respectively) was constructed. After removing the low-quality reads, an average of 78 776 032, 90 032 505, 74 915 467, 83 592 932, 78 553 496, and 86 599 589 clean reads were obtained respectively and the number of clean bases was 11.82G, 13.50G, 11.24G, 12.54G, 11.78G and 12.99G. The average GC content was 53.01 %, 53.46 %, 52.09 %, 51.83 %, 52.36 % and 52.59 %; Q20 averages were 97.22 %, 97.84 %, 97.65 %, 98.07 %, 97.91 % and 97.93 %; and Q30 averages were 92.02 %, 93.21 %, 92.93 %, 93.79 %, 93.39 % and 93.40 %, indicating that the transcriptome sequencing data met the requirements of subsequent experiments (Figs. 3–7).

### Functional annotation of unigenes

Gene annotation and functional enrichment analyses, including Gene Ontology (GO) and Kyoto Encyclopedia of Genes and Genomes (KEGG) biological pathways, were conducted. GO analysis showed that the genes could be further divided into 38 subclasses in three broad categories: molecular functions, cellular components, and biological processes. Cellular processes, metabolic processes, single-organism processes, catalytic activity and binding were the most enriched subclasses.

### Screening and analysis of differentially expressed genes

A comparison of the genes of the three BR low-temperature treatment groups (B1, B2 and B3) and the three low-temperature control groups (D1, D2 and D3) showed that as temperature decreased. The number of upregulated genes showed a trend of first decreasing and then increasing. The number of downregulated genes showed a gradual downward trend. Using |log2(FoldChange)| ≥ 1 and padj ≤ 0.05 as the screening criteria, a total of 21 372 differentially expressed genes (DEGs) were screened. Among the DEGs, 8198 genes were upregulated and 7104 genes were downregulated in B1VSD1 tillering node samples; 1930 genes were upregulated and 1456 genes were downregulated in B2VSD2 tillering node samples; and 2102 genes were upregulated and 582 genes were downregulated in B3VSD3 tillering node samples.

### GO enrichment analysis

To understand the functions of differentially expressed genes (DEGs), GO enrichment analysis was performed on the tillering nodes of wheat in the BR-treated group and the control group under different levels of low-temperature stress. All DEGs were classified into three main functional categories: 171 B1 vs D1, 56 B2 vs D2, and 56 B3 vs D3 GO terms.

In B1 vs D1, the number of GO terms for biological processes, cellular components, and molecular functions was 82, 35 and 58, respectively. Under biological processes, DEGs were significantly enriched in the terms photosynthesis, DNA replication, and ribosome biogenesis. Under the cellular component category, the DEGs were significantly enriched in the terms thylakoid, thylakoid part and photosynthetic membranes. Under molecular functions, the DEGs were significantly enriched in the terms 2 iron, 2 sulphur cluster binding, carbon–oxygen lyase activity and oxidoreductase activity, acting on single donors with the incorporation of molecular oxygen.

In B2 vs D2, the number of GO terms for biological processes, cellular components, and molecular functions was 19, 6 and 31, respectively. Under the biological process category, the DEGs were significantly enriched in the terms cellular carbohydrate metabolic process, disaccharide metabolic process, and oligosaccharide metabolic process; under the cellular component category, the DEGs were significantly enriched in the terms extracellular region, apoplast, and extracellular matrix, and under molecular functions, the DEGs were significantly enriched in the terms protein heterodimerization activity, chitin binding and primary active transmembrane transporter activity.

At B3 vs D3, the number of GO terms in biological processes, cellular components, and molecular functions was 1, 16 and 39, respectively. Under biological processes, the DEGs were significantly enriched in the terms photosynthesis, cell redox homeostasis, and response to oxidative stress. Under cellular component, the DEGs were significantly enriched in the terms thylakoid, thylakoid part, and photosynthetic membranes; under molecular functions, the DEGs were significantly enriched in the terms fructose 1, 6-bisphosphate-1-phosphatase activity, oxidoreductase activity, acting on single donors with incorporation of molecular oxygen, incorporation of one atom of oxygen (internal monooxygenases or internal mixed function oxidases) and FMN binding.

### KEGG enrichment analysis of DEGs

To functionally classify the brassinolide-treated wheat seedlings under different low temperature stresses and to analyse the pathway enrichment analysis of DEGs, KEGG pathway enrichment analysis was performed.

**Figure 8.**
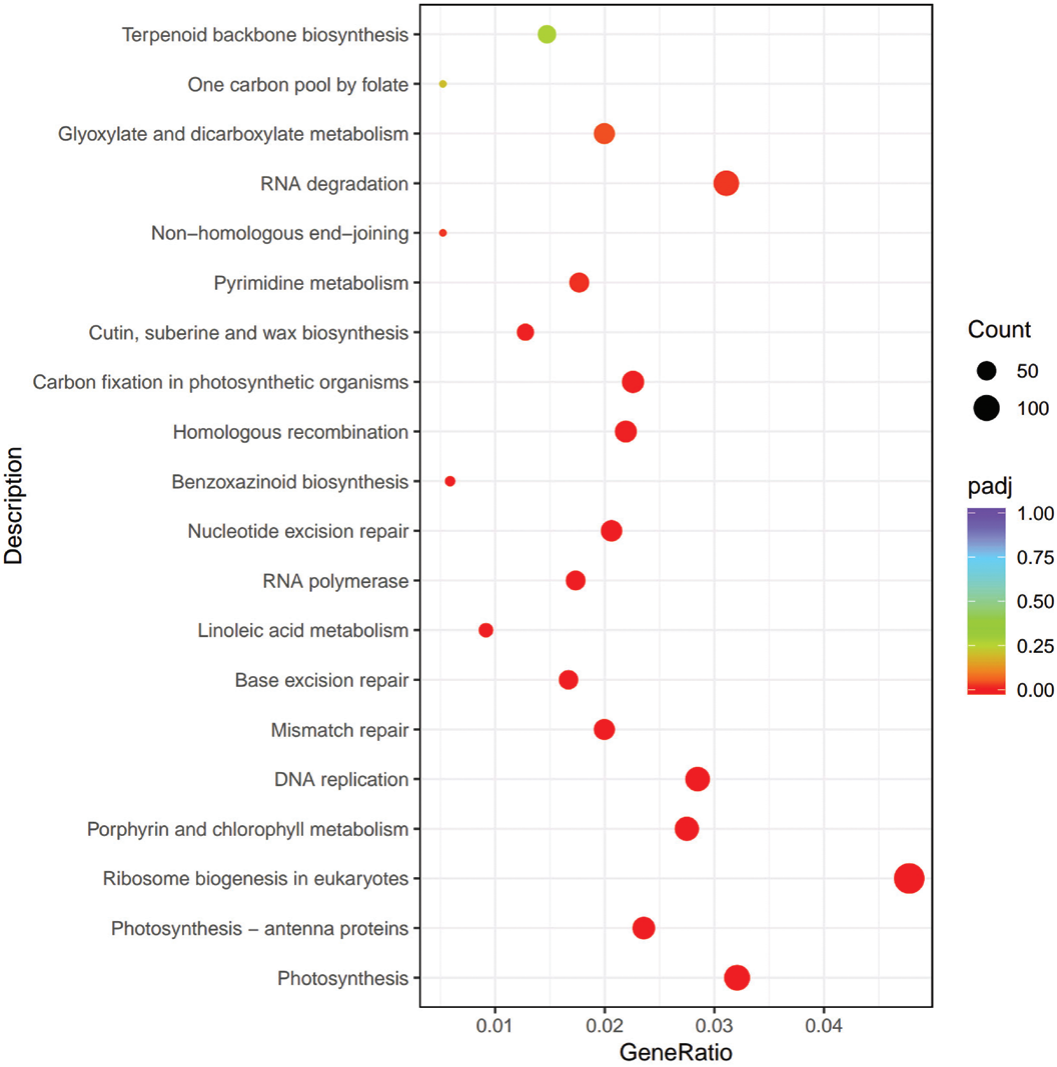
B1 vs D1 KEGG pathway enrichment analysis of differentially expressed genes (DEGs).

**Figure 9.**
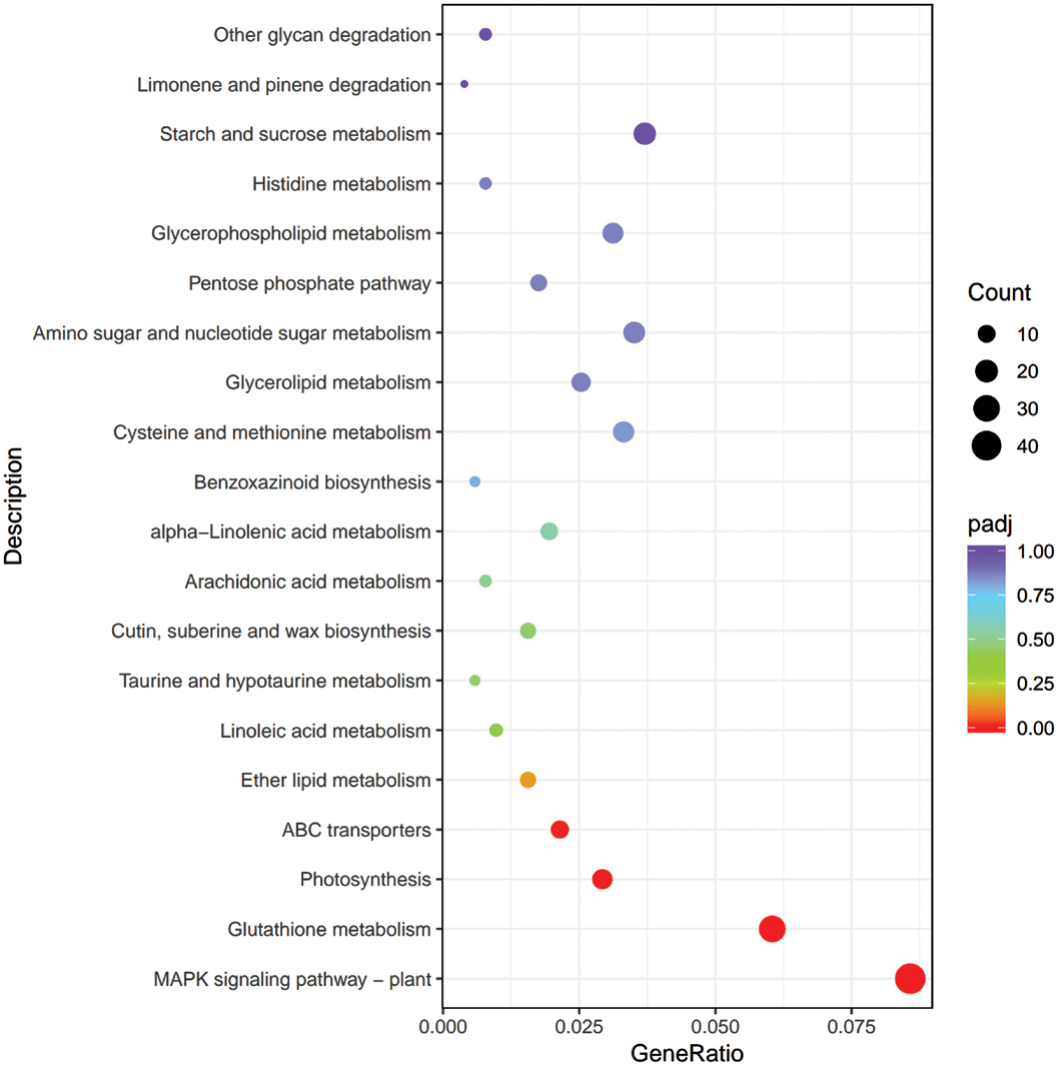
B2 vs D2 KEGG pathway enrichment analysis of differentially expressed genes (DEGs).

In B1VSD1, 3056 DEGs were assigned to 119 KEGG pathways. The three branches of environmental information, genetic information and metabolism were significantly enriched (*P* < 0.05), among which metabolism-rich DEGs were dominant. Figure 8 shows the 20 pathways most involved in DEGs in the response of wheat seedlings to low-temperature stress, among which photosynthesis, photosynthesis-antenna proteins, and ribosome biogenesis in eukaryotes were the most representative pathways (Fig. 9).

In B2 vs D2, 513 DEGs were assigned to 92 pathways, and environmental information processing and metabolism were the most representative pathways.

**Figure 10.**
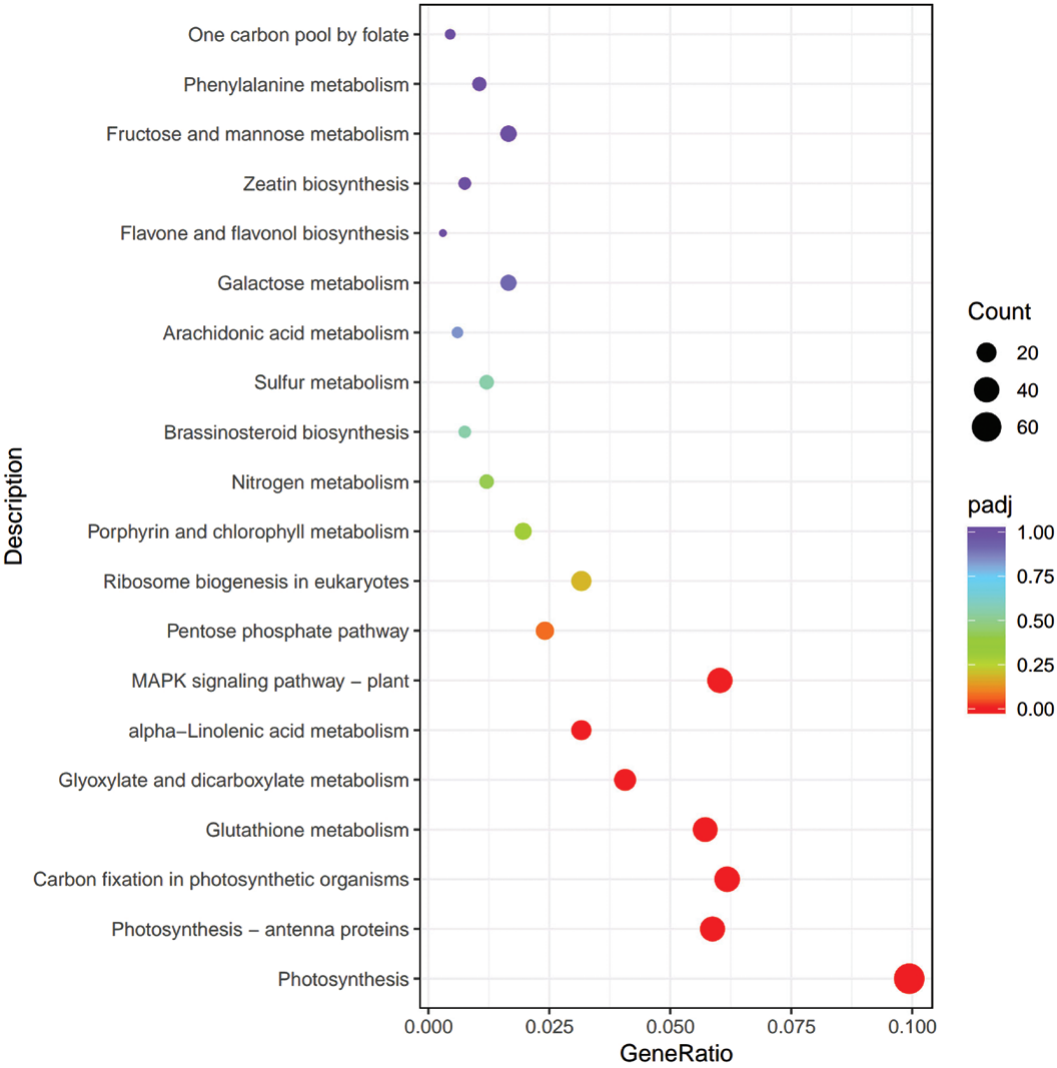
B3 vs D3 KEGG pathway enrichment analysis of differentially expressed genes (DEGs).

In B3 vs D3, a total of 664 DGEs were assigned to 100 pathways, of which metabolism was the dominant pathway, and Fig. 10 shows the 20 pathways in which DEGs were most involved.

### Key regulatory pathways of brassinolide in winter wheat

**Figure 11.**
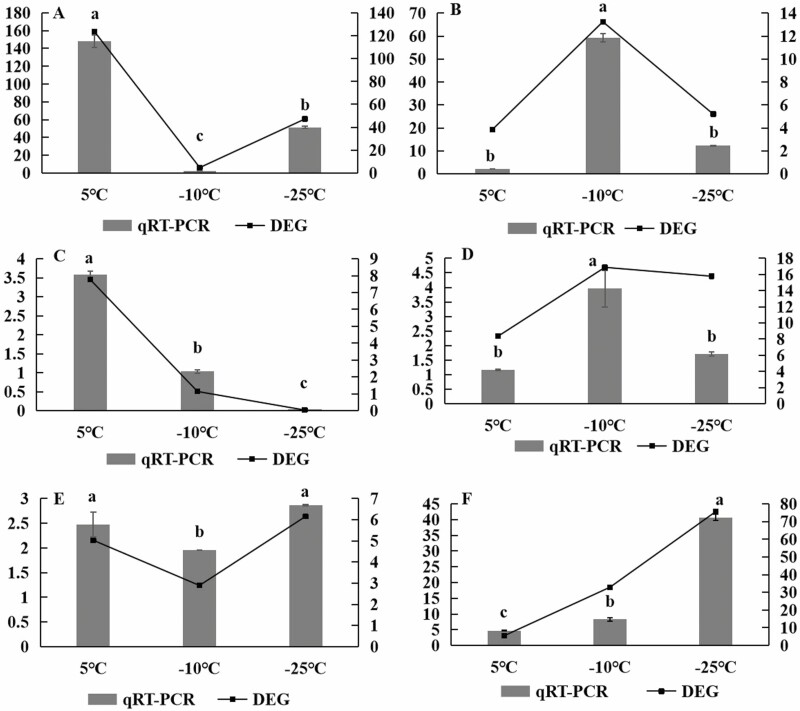
qRT-PCR validation of cold resistance-related genes in wheat. Note: A: ERF003-like; B: REVEILLE 6-like; C: synthase-like; D: BRI1-like; E: RF2a-like; F: WRKY28-like. Error bars represent the standard deviation of the mean relative expression levels of qRT-PCR (left *y*-axis). Dotted line plots represent FPKM values for RNA-Seq (right y-axis) (p < 0.05).

Brassinolide is one of the most active and broad-spectrum plant growth hormones recognized internationally so far. Under low temperature stress, brassinolide regulates winter wheat through photosynthesis, photosynthesis antenna proteins and the MAPK signalling pathway. Photosynthesis is the main regulatory pathway, indicating that brassinolide can promote the metabolism of winter wheat under low temperature. The key genes involved in regulation are 90A4-like (LOC123120303) and synthase-like (LOC123121528) (Fig. 11).

### DEG validation by qRT-PCR

To verify the reliability of the identified DEGs in the effect of brassinolide on Dongnong dongmai 1 at low temperatures, six genes were selected at different temperatures, and their expression was detected by qRT-PCR analysis (three biological replicates per sample). The results showed excellent agreement between the expression levels of the six genes analysed by qRT-PCR and the expression levels of the six genes detected by RNA-seq. Compared to the control group at 5 °C, the difference was significant, indicating that BR plays a regulatory role in a low-temperature environment, and demonstrating that the transcriptome sequencing results have high accuracy and reliability.

## Discussion

### Effects of low temperature and brassinolide on plants

Low temperature stress affects the productivity of cereals and other plants, most importantly in temperate regions. Plants respond differently to freezing (<5 °C) and cooling (0–15 °C). Cold adaptation and acquisition of freeze tolerance are achieved by exposure to cool, non-freezing temperatures (Thomashow *et al.* 1999; Levitt 1980; Laudencia-Chingcuanco *et al.* 2011; Fowler *et al.* 1979). This process increases freezing tolerance and is associated with complex biochemical and physiological changes (Thomashow *et al.* 1999; Levitt 1980; Laudencia-Chingcuanco *et al.* 2011; Fowler *et al.* 1979; Ouellet *et al.* 2001). These changes are mediated by the differential expression of multiple genes (Chinnusamy *et al.* 2007; Monroy *et al.* 2007; Shinozaki *et al.* 2000; Kosmala *et al.* 2009). Experimental studies have shown that these genes are induced by cold or relative dehydration caused by cold stress (Gilmour *et al.* 2000).

Several cold-regulated genes have been identified in gene expression studies. Many cold-regulated genes have been assigned specific functions, such as transcription factors (TFs) that act as regulators in cold adaptation or effector molecules that mitigate potential damage caused by cold stress. However, the specific molecular functions of many cold-responsive genes have not been investigated and their roles in cold adaptation remain unclear (Tsuda *et al.* 2000). Deciphering the low-temperature (LT) response mechanism and the specific roles of key genes involved in cold stress signalling are critical for the development of cold-tolerant crops. Analysis of gene expression data showed that, under specific conditions, a plant’s encoded protein can have a significant impact on the plant phenotype. The identified genes provide a basis for establishing targeted functional markers that can be used for assisted breeding (Jain *et al.* 2009; Kole *et al.* 2015). Differentially expressed genes play key roles in low-temperature tolerance in wheat.

To gain insight into the molecular mechanisms involved in wheat seedling growth under low temperature and brassinolide stress, RNA-seq analysis was performed. A total of 21 372 differentially expressed genes were identified in wheat seedlings, and 15 302 (8198 upregulated and 7104 downregulated) differentially expressed genes (DEGs) were identified in B1 vs D1; 3386 (1930 upregulated and 1456 downregulated) differentially expressed genes (DEGs) were identified in B2 vs D2; and 2684 (2102 upregulated and 582 downregulated) differentially expressed genes (DEGs) were identified in B3 vs D3 group. These results indicate that gene expression in wheat undergoes severe changes with BR treatment under low-temperature stress.

GO enrichment analysis showed that the DEG enrichment ratio was highest when the temperature was 5 °C. Some researchers believe that the transcripts of differentially expressed genes in these processes may play a crucial role in plant adaptation to cold environments. For example, in Arabidopsis, hundreds of genes are differentially expressed in response to cold stress (Novillo *et al.* 2004; Seki *et al.* 2002; Provart *et al.* 2003; Vogel *et al.* 2004; Zhang *et al.* 2009). In temperate regions, including perennial rye (Zhang *et al.* 2009), barley (Svensson *et al.* 2006) and wheat (Skinner *et al.* 2015; Vítámvás *et al.* 2007; Houde *et al.* 1995; Christov *et al.* 2008), the expression of many genes is also altered by cold stress.

The cold resistance of plants was improved by BR treatment. In GO enrichment analysis, DEGs are involved in photosynthesis, and BR is also an important factor in regulating photosynthesis; for example, it can affect PSII efficiency (Sadura *et al.* 2018; Holá *et al.* 2011; Fariduddin *et al.* 2014; Li *et al.* 2021). The occurrence and physiological activity of BR in grains are discussed in more detail in Janeczko (2019) and Janeczko *et al.* (2019). On the other hand, a review by Sadura and Janeczko (Sadura *et al.* 2018) discussed the role of BR in plant responses to temperature stress. However, relatively few studies have been conducted on BR and temperature stress (Sadura *et al.* 2018). The results showed that in GO enrichment analysis, genes related to lyase activity, protein heterodimerization activity, and membrane protein complex were upregulated, which may provide the necessary substances and energy for wheat seedlings to resist or adapt to low-temperature stress.

KEGG analysis showed that under low-temperature and BR stress, DEGs were mainly involved in photosynthesis, photosynthesis antenna proteins and ribosome biogenesis in eukaryotes, and that these pathways were mainly related to environmental information processing and metabolism. Photosynthesis is an important part of green plants’ life cycle, providing the necessary energy for plant metabolism (Chen *et al.* 2014; Zhang *et al.* 2016), and among abiotic stresses, temperature stress poses a particularly serious problem in plant production (Zhang *et al.* 2022; Hasanuzzaman *et al.* 2012). Liu *et al.* (2016) demonstrated a link between BR biosynthesis and chlorophyll biosynthesis in cereals. In cold adapted, frost-tolerant winter rye (variety Dańkowskie Złote) exogenously treated with 24-epibrassinolide, the energy flow from the photosynthetic antenna to the electron transport chain was more efficient than that in untreated controls (Pociecha *et al.* 2016). Dou *et al.* (2021) upregulated photosynthesis-related DEGs, suggesting that BR may have a positive role in regulating photosynthesis under cold stress conditions. Jin *et al.* (2022) showed through KEGG enrichment annotation that 26 genes are involved in the BR signal transduction pathway, and that spraying BR on tea can promote the upregulation of genes involved in the BR signal transduction pathway and promote tea cell division, theanine acid synthesis, and increased expression of genes associated with cold tolerance. These results showed that the material and energy migration of wheat seedlings may be enhanced under low-temperature stress, and this adaptation is significant for the resistance or adaptability of wheat to low-temperature environments.

In conclusion, a transcriptomic approach was used to compare brassinolide mRNA expression in wheat tillering nodes at low temperatures. A total of 21 372 differentially expressed genes were identified in the tillering nodes, and 15 302 (8198 upregulated and 7104 downregulated) differentially expressed genes (DEGs) were identified in B1 vs D1; 3,386 (1930 upregulated and 1456 downregulated) differentially expressed genes (DEGs) were identified in B2 vs D2; and 2684 (2102 upregulated and 582 downregulated) differentially expressed genes (DEGs) were identified in the B3 vs D3. Further annotation and analysis showed that the DEGs were mainly enriched in four major pathways, which were mainly related to metabolic processes. This indicates that, at low temperatures, brassinolide can enhance the metabolism of winter wheat and generate more energy and substances to resist or adapt to an unfavourable environment. Overall, these results expand our understanding of the molecular mechanism of brassinolide in winter wheat under low-temperature stress and provide a valuable resource for further research on the effect of brassinolide on Dongnong dongmai 1 under low-temperature stress.

## Data Availability

The RNA-seq data that support the findings of this study are openly available in NCBI Sequence Read Archive (SRA) database at (https://www.ncbi.nlm.nih.gov) with the accession number (PRJNA914476).
